# Imaging in gynecological disease (32): clinical and ultrasound characteristics of early abdominal ectopic pregnancy

**DOI:** 10.1002/uog.70342

**Published:** 2026-09-20

**Authors:** L. Berg, L. V. de Braud, E. Bean, M. Lomheim, L. Savelli, D. Jurkovic

**Affiliations:** ^1^ EGA Institute for Women's Health, Faculty of Population Health Sciences University College London (UCL) London UK; ^2^ Barking Havering and Redbridge University Hospitals NHS Trust London UK; ^3^ Mandal Spesialistsenter Mandal Norway; ^4^ Obstetrics and Gynaecology Unit, Morgagni‐Pierantoni Hospital, Forlì & University of Bologna School of Medicine Bologna Italy

**Keywords:** abdominal pregnancy, assisted reproductive technology, diagnosis, ultrasound

## Abstract

**Objectives:**

To compare clinical and sonographic characteristics of first‐trimester abdominal ectopic pregnancy (AEP) with those of tubal ectopic pregnancy (TEP), and to define ultrasound‐based diagnostic criteria for AEP.

**Methods:**

This was a retrospective case–control study comparing cases of AEP diagnosed at four centers with controls diagnosed with TEP, between May 2008 and May 2025. AEP was defined as a pregnancy implanted within the peritoneal cavity outside the uterus, ovaries and Fallopian tubes. We compared demographics, clinical and ultrasound findings and clinical outcomes between the groups. We identified the key ultrasound features of first‐trimester AEP and used these to develop refined diagnostic criteria.

**Results:**

During the study period, 19 cases of AEP were identified and compared to 57 controls with TEP. The groups were matched for age, parity and gestational age at presentation. Compared with TEP, AEP was more likely to occur in pregnancies conceived via *in‐vitro* fertilization (6/19 (32%) *vs* 5/57 (9%); odds ratio (OR) 4.80 (95% CI, 1.27–18.21); *P* = 0.02; adjusted OR (aOR), 5.02 (95% CI, 1.24–20.25); *P* = 0.02) and patients with AEP were less likely to present with vaginal bleeding (5/18 (28%) *vs* 40/57 (70%); OR, 0.16 (95% CI, 0.05–0.53); *P* = 0.003; aOR, 0.17 (95% CI, 0.05–0.64); *P* = 0.009). With respect to clinical outcomes, patients with AEP were more likely to have significant blood loss (≥ 500 ml) at surgery (4/16 (25%) *vs* 1/36 (3%); OR, 11.67 (95% CI, 1.18–114.90); *P* = 0.04) and require blood transfusion (4/19 (21%) *vs* 0/56 (0%); *P* = 0.003). Four diagnostic ultrasound criteria for AEP were identified: (1) the presence of a gestational sac or trophoblast separate from the uterus and ovaries; (2) the absence of a hypoechogenic tissue layer surrounding the pregnancy; (3) a negative ‘sliding organs’ sign; and (4) trophoblastic blood flow arising from the peritoneum detected on color Doppler examination.

**Conclusions:**

Assisted conception is a risk factor for a pregnancy implanting into the peritoneal cavity. Early ultrasound diagnosis of AEP is important to reduce the risk of major intra‐abdominal bleeding, which is significantly higher in AEP compared with TEP. © 2026 The Author(s). *Ultrasound in Obstetrics & Gynecology* published by John Wiley & Sons Ltd on behalf of International Society of Ultrasound in Obstetrics and Gynecology.

## INTRODUCTION

### Aim

The aim of this study was to identify risk factors and propose novel ultrasound criteria for the diagnosis of abdominal ectopic pregnancy (AEP).

### Background

#### Epidemiology

The European Society of Human Reproduction and Embryology (ESHRE) define AEP as a pregnancy that implants within the peritoneal cavity, outside the uterus, ovaries and Fallopian tubes^1^. Overall, ectopic pregnancies account for 1–2% of all pregnancies^2^. While 90% of ectopic pregnancies occur in the Fallopian tubes, AEP is estimated to account for approximately 1% of ectopic pregnancies, or 1/10 000 pregnancies overall^3^, ^4^. The terms ‘early’ and ‘late’ have been used to distinguish between AEP diagnosed before and after 20 weeks' gestation, respectively^5^. With respect to pathogenesis, it has been hypothesized that AEP results from failure of the fimbrial end of the Fallopian tube to capture the oocyte that is released at ovulation, resulting in fertilization by sperm in the peritoneal cavity (‘primary AEP’) or peritoneal implantation of the conceptus following spontaneous expulsion from the Fallopian tube or after tubal rupture (‘secondary AEP’)^6^. Some authors have argued that all AEPs originate in the Fallopian tube^7^, however reports of AEP following bilateral salpingectomy^8^ do not support this hypothesis. In individual cases, the exact pathophysiological mechanism is not always clear, but this has no clinical impact.

#### Microscopy

Microscopically, AEP is diagnosed by the presence of villous tissue alongside peritoneal tissue, which confirms that the pregnancy implanted into the peritoneum. However, histopathological analysis is not essential for diagnosis and is not possible in cases managed without surgery. Furthermore, if a small section of tissue is analyzed with the sole aim of confirming the presence of trophoblast, the presence of peritoneal tissue may not be assessed.

#### Macroscopy

The macroscopic appearance of AEP varies from a heterogeneous solid lesion to a morphologically normal pregnancy, formed of a gestational sac containing embryonic structures. Various sites of implantation within the abdomen have been reported. The most common locations of AEP implantation are the pouch of Douglas, uterovesical fold and the serosa of the uterus and broad ligament. Other sites of implantation include the omentum, bowel, appendix, liver, spleen and even the retroperitoneum^9^. The location of implantation and source of blood supply may significantly impact the ease and timing of diagnosis and the treatment options available.

#### Clinical symptoms and prognosis

There are several factors that make the diagnosis of AEP challenging. The first is its rarity, which limits familiarity with the condition, even among experienced clinicians. Second, the clinical symptoms of vaginal bleeding and abdominal pain at presentation may be mild or absent entirely^10^, ^11^, ^12^, ^13^. This means that there is considerable crossover with the clinical presentation of more common early‐pregnancy complications, as well as with that of healthy pregnancies. Every early‐pregnancy ultrasound examination should include assessment of the cervix, uterine cavity and myometrium (including Cesarean or any other uterine scar, if present). The examination should also include the interstitial segment of the Fallopian tubes, adnexa and ovaries. If no pregnancy is identified after comprehensive assessment of these structures, the possibility of AEP should be considered. Given that most AEPs implant in the pouch of Douglas, uterovesical fold or the serosa of the uterus and broad ligament, the examination should be extended to include these areas of the pelvis.

The prognosis for AEP is varied and depends on gestational age at diagnosis. As with other types of pregnancy, hemorrhage that disrupts the implantation site may lead to early pregnancy failure and spontaneous resolution. Alternatively, such hemorrhage may result in significant internal bleeding, requiring urgent surgical intervention^10^. Undiagnosed early AEP can develop to advanced gestations and ultimately result in a live birth, although this is associated with significant risks of maternal and fetal morbidity and mortality. Reported maternal mortality in AEP ranges between 0.5% and 20%, which is 90‐times higher than in normally sited (eutopic) pregnancies and nearly 8‐times higher than in other types of ectopic pregnancy^3^. Aside from acute hemorrhage, other maternal risks of AEP include infection, pre‐eclampsia/eclampsia, amniotic fluid embolism and intestinal obstruction^5^. Reported perinatal mortality rates range from 45% to 90%, and the risks of oligohydramnios, intrauterine growth restriction and fetal malformations are increased^3^, ^12^.

Diagnosis of AEP in the first trimester is critical for patient safety. However, most reported cases of AEP are diagnosed surgically, with rates of sonographic diagnosis made outside of an emergency setting reported to vary between 10% and 57%^2^, ^5^, ^8^, ^9^. Sonographic detection rates for tubal ectopic pregnancy (TEP) can approach 100% in dedicated early pregnancy centers^14^, suggesting that detection rates for early AEP could also be improved.

## METHODS

This was a case–control study including consecutive patients with AEP and frequency‐matched controls with TEP. Cases were identified predominantly from a cohort of patients attending the Early Pregnancy Unit at University College London Hospital (UCLH), London, UK, between May 2008 and May 2025, with several cases included from other centers (Homerton University Hospital NHS Foundation Trust, London, UK; Barking, Havering and Redbridge University Hospitals NHS Trust, London, UK; Mandal Spesialistsenter, Mandal, Norway). Sample size was dictated by the total number of consecutive cases of AEP available across the study period. We sought advice from the National Health Service Health Research Authority and the Joint Research Office at UCLH regarding ethical approval, who advised that formal ethical approval was not required for this study, since the data had already been collected as part of routine care and was anonymized and analyzed within the clinical care team. This study was registered with the Research Registry (unique identifying number: researchregistry11133).

All patients presenting to our center with a positive urine pregnancy test are assessed clinically and offered a transvaginal and/or transabdominal ultrasound scan using high‐resolution equipment (Voluson 730 and E8 Expert; GE Healthcare, Zipf, Austria). In this study, ultrasound assessments were performed by experienced (European Federation of Societies for Ultrasound in Medicine and Biology (EFSUMB) Level‐II) operators, with the diagnosis confirmed by expert (EFSUMB Level‐III) operators^15^. AEP was diagnosed when a pregnancy was located in the peritoneal cavity, separate to the uterus, Fallopian tubes and ovaries. Once an extrauterine ectopic pregnancy had been identified, its relation to the surrounding structures was evaluated via bimanual palpation with a transvaginal probe to establish mobility relative to surrounding tissues. Color Doppler examination was used routinely to assess the blood supply to the pregnancy, to provide information about the exact site of implantation. The ultrasound morphology of the pregnancy was classified according to criteria described by Dooley *et al*.^14^. Management was individualized according to clinical symptoms, gestational age at diagnosis, evidence of embryonic cardiac activity, presence of hemoperitoneum, serum human chorionic gonadotropin (hCG) levels and patient preference. The hCG resolution time was defined as the number of days from presentation to the time during follow‐up at which the serum hCG level declined to a non‐pregnant level (< 20 IU/L). Patient demographic and clinical data and ultrasound images were stored in our clinical database (Viewpoint Version 5; Bildverargeritung GmbH, Munich, Germany). At the primary center (UCLH), all cases of AEP were scanned by expert sonographers. Subsequently, the images were reviewed, or patients were re‐examined, by a second senior expert operator, who confirmed the diagnosis. All cases of AEP included from other clinical sites were confirmed histologically after surgical treatment. Cases were compared with controls diagnosed with TEP during the same time period at the primary study center, in a 1:3 ratio. The two groups were frequency‐matched with respect to age, parity and gestational age at presentation.

Parametric variables are expressed as mean ± SD. Non‐parametric variables are expressed as median (interquartile range (IQR)). Continuous variables were compared using the unpaired *t*‐test if the data were normally distributed or the Mann–Whitney *U*‐test if non‐normally distributed. Categorical variables were compared using the chi‐square test or Fisher's exact test. Given the binary nature of the outcome, all subsequent analysis was performed using logistic regression. Odds ratios (ORs) with 95% CI were calculated, and *P* < 0.05 was considered statistically significant. Potential risk factors for AEP were analyzed in two stages. Initially, the association between each risk factor and the outcome was examined separately in a series of univariable analyses. Subsequently, the joint association between the risk factors and case/control status was assessed via multivariable analysis. To restrict the number of variables in this second stage of the analysis, only factors with a univariable *P* of < 0.15 were included. A backwards selection procedure was used to retain only the significant variables in the final model. Clinical outcomes associated with AEP were also identified in regression analysis. Initially, the outcome was compared between the two groups without considering any other factors (unadjusted analysis). Subsequently, differences in outcomes between the two groups were adjusted for the risk factors for AEP identified in the previous multivariable analysis, with the exception of operative blood loss ≥ 500 ml, for which adjusted analysis was not deemed appropriate owing to the low event rate. Binary outcomes were compared using logistic regression, ordinal outcomes were compared using ordinal logistic regression and continuous outcomes were analyzed using linear regression. The outcome variable was analyzed on the original scale of measurement if normally distributed, or on the log scale if found to follow a positively skewed distribution. Missing data were handled using complete‐case analysis. Ultrasound images of AEPs were reviewed retrospectively by an expert (EFSUMB Level‐III)^15^ operator (D.J.) in an attempt to identify morphological features that could be used to differentiate AEP from other types of extrauterine ectopic pregnancies.

## RESULTS

During the study period, 19 cases of AEP were diagnosed and matched to 57 controls with TEP. 15/19 cases of AEP were managed at our center, and 4/19 cases were contributed from the three other centers. The baseline characteristics of cases and controls are shown in Table 1.

**Table 1:** Baseline characteristics of cases of abdominal ectopic pregnancy (AEP) and controls with tubal ectopic pregnancy (TEP)

Characteristic	AEP (*n* = 19)	TEP (*n* = 57)	*P*
Maternal age	33.1 ± 4.8	32.6 ± 4.8	0.73
Parity			0.98
0	11 (58)	33 (58)	
1	5 (26)	16 (28)	
2+	3 (16)	8 (14)	
GA at presentation (days)	46 (42–55)	49 (41–54)	0.88

Data are given as mean ± SD, *n* (%) or median (interquartile range). GA, gestational age.

### Risk factors

Potential risk factors for the development of AEP were assessed in univariable and multivariable analyses. The results of the univariable analysis are shown in Table 2. The univariable analysis suggested possible positive associations between AEP and conception via *in‐vitro* fertilization (IVF), endometriosis and previous ectopic pregnancy and possible negative associations with previous termination of pregnancy (TOP) and previous surgical management of miscarriage (SMM)/surgical TOP (STOP). The results reached statistical significance for mode of conception (6/19 (32%) cases of AEP conceived via IVF *vs* 5/57 (9%) controls with TEP concieved via IVF; OR, 4.80 (95% CI, 1.27–18.21); *P* = 0.02), but not for the other factors.

**Table 2:** Potential risk factors associated with abdominal ectopic pregnancy (AEP) compared with tubal ectopic pregnancy (TEP)

Risk factor	AEP (*n* = 19)	TEP (*n* = 57)	Odds ratio (95% CI)	*P*
Ethnicity				0.15
White	10/14 (71)	19/35 (54)	1 (reference)	
Black	0/14 (0)	8/35 (23)	NE	
Mixed/other	4/14 (29)	8/35 (23)	NE	
BMI*				1.00
Underweight/normal	3/9 (33)	10/30 (33)	1 (reference)	
Overweight/obese	6/9 (67)	20/30 (67)	1.00 (0.21–4.86)	
Previous miscarriage	7 (37)	17 (30)	1.37 (0.46–4.09)	0.57
Previous TOP	1 (5)	15 (26)	0.16 (0.02–1.27)	0.08
Previous ectopic pregnancy	2 (11)	1 (2)	6.59 (0.56–77.19)	0.13
Previous SMM/STOP	1 (5)	15 (26)	0.16 (0.02–1.27)	0.08
Previous Cesarean section	3 (16)	6 (11)	1.59 (0.36–7.11)	0.54
Other previous pelvic surgery	3 (16)	5 (9)	1.95 (0.42–9.07)	0.39
Endometriosis	5 (26)	5 (9)	3.71 (0.94–14.66)	0.06
Mode of conception				0.02
Spontaneous	13 (68)	52 (91)	1 (reference)	
IVF	6 (32)	5 (9)	4.80 (1.27–18.21)	

Data are given as *n*/*N* (%) or *n* (%), unless stated otherwise.

* Body mass index (BMI) ≤ 24.9 kg/m^2^ was considered underweight/normal, and > 24.9 kg/m^2^ was considered overweight/obese. IVF, *in‐vitro* fertilization; NE, not estimable; SMM, surgical management of miscarriage; STOP, surgical termination of pregnancy; TOP, termination of pregnancy.

The results of the multivariable analysis are shown in Table 3. The multivariable analysis showed some evidence that previous ectopic pregnancy, previous TOP and conception via IVF were associated with AEP. The results for conception via IVF were significant (adjusted OR (aOR), 5.02 (95% CI, 1.24–20.25); *P* = 0.02), while the results for the other two variables did not reach statistical significance. However, owing to the relatively large effects of these two variables, they were retained in the final model. After adjusting for these three variables, there were no additional effects of either previous SMM/STOP or endometriosis upon the case/control status.

**Table 3:** Multivariable association between independently associated risk factors and abdominal ectopic pregnancy (AEP)

Variable	aOR (95% CI)	*P*
Previous TOP	0.13 (0.01–1.34)	0.09
Previous ectopic pregnancy	16.0 (0.87–296.13)	0.06
Conception via IVF	5.02 (1.24–20.25)	0.02

aOR, adjusted odds ratio; IVF, *in‐vitro* fertilization; TOP, termination of pregnancy.

### *In‐vitro* fertilization

All patients with AEP and TEP who conceived via IVF had undergone frozen‐embryo transfer. 3/6 (50%) cases of AEP conceived via IVF underwent single‐embryo transfer and the other 50% underwent double‐embryo transfer. 3/3 (100%) controls with TEP conceived via IVF for whom the number of embryos transferred was known had undergone single‐embryo transfer (this was unknown for 2/5 controls with TEP that conceived via IVF).

### Clinical presentation

Clinical symptoms and ultrasound characteristics at presentation of cases with AEP and controls with TEP are shown in Table 4. The unadjusted univariable analysis suggested a negative association between AEP and vaginal bleeding (5/18 (28%) cases *vs* 40/57 (70%) controls; OR, 0.16 (95% CI, 0.05–0.53); *P* = 0.003) and a positive association between AEP and higher serum hCG level at diagnosis (2041 (IQR, 1087–4464) IU/L *vs* 776 (IQR, 225–2724) IU/L; OR, 2.40 (95% CI, 1.04–5.54); *P* = 0.04). Only the negative association between AEP and vaginal bleeding remained statistically significant in the adjusted analysis (aOR, 0.17 (95% CI, 0.05–0.64); *P* = 0.009).

**Table 4:** Clinical and ultrasound features at presentation of cases of abdominal ectopic pregnancy (AEP) and controls with tubal ectopic pregnancy (TEP)

Feature	AEP (*n* = 19)	TEP (*n* = 57)	OR (95% CI)	*P*	Adjusted OR (95% CI)*	*P*
Abdominal pain	10/18 (56)	36 (63)	0.73 (0.25–2.13)	0.56	0.92 (0.28–3.08)	0.90
Vaginal bleeding	5/18 (28)	40 (70)	0.16 (0.05–0.53)	0.003	0.17 (0.05–0.64)	0.009
Ultrasound morphology				0.12		0.16
Live embryo	4/17 (24)	5/56 (9)	1 (reference)		1 (reference)	
Other	13/17 (76)	51/56 (91)	3.14 (0.74–13.37)		2.91 (0.65–13.13)	—
Hemoperitoneum			1.97 (0.69–5.67)§	0.21	2.02 (0.62–6.59)§	0.24
Absent	10/18 (56)	41 (72)	—		—	
Mild	5/18 (28)	10 (18)	—		—	—
Moderate	2/18 (11)	4 (7)	—		—	
Severe	1/18 (6)	2 (4)	—		—	
Serum hCG at diagnosis (IU/L)†	2041 (1087–4464)	776 (255–2724)	2.40 (1.04–5.54)	0.04	1.70 (0.66–4.36)	0.26
Serum progesterone at diagnosis (nmol/l)‡	22 (4–97)	16 (6–30)	1.42 (0.64–3.14)	0.38	1.70 (0.66–4.37)	0.26

Data are given as *n*/*N* (%), median (interquartile range) or *n* (%), unless stated otherwise.

* Adjusted for previous termination of pregnancy, previous ectopic pregnancy and mode of conception.

† Data unavailable for 1/19 patients with AEP and 6/57 patients with TEP.

‡ Data unavailable for 2/19 patients with AEP and 7/57 patients with TEP.

§ Odds ratio (OR) per one‐category increase in severity. hCG, human chorionic gonadotropin.

### Diagnosis

One case of AEP was referred to our center for management of residual ectopic pregnancy after surgical diagnosis at another center, therefore the diagnosis was already known. Of the remaining cases, AEP was correctly diagnosed on ultrasound examination in 12/18 (67%) cases. Ultrasound diagnosis of AEP was made at the initial examination in 10/12 (83%) cases. In the other two cases, the diagnosis of AEP was made during follow‐up scans, after initial ultrasound diagnosis of pregnancy of unknown location and TEP, respectively. In 6/18 (33%) cases, AEP was diagnosed surgically. In 5/6 (83%) of these cases, a previous ultrasound diagnosis of TEP had been made and in 1/6 (17%) cases, the ultrasound diagnosis was suspected ruptured ectopic pregnancy, owing to the finding of severe hemoperitoneum in the absence of a visible pregnancy.

Pregnancy morphology varied from an inhomogeneous solid swelling in 7/17 (41%) cases to an empty gestational sac in 3/17 (18%) cases, a gestational sac containing a yolk sac in 2/17 (12%) cases, a gestational sac containing an embryo without cardiac activity in 1/17 (6%) cases and a gestational sac containing a live embryo in 4/17 (24%) cases. The morphology was unknown for two cases in which the pregnancy could not be visualized sonographically prior to surgery.

In order to accurately locate the AEP within the pelvis, the relation of the pregnancy to pelvic landmarks was assessed. In our cohort, 10/19 (53%) pregnancies were implanted within the pouch of Douglas (Figure 1), 3/19 (16%) were implanted within the uterovesical fold (Figure 2), 5/19 (26%) were implanted at the pelvic sidewall (Figure 3) and 1/19 (5%) cases was identified at surgery to be implanted on the omentum.

**Figure 1:**
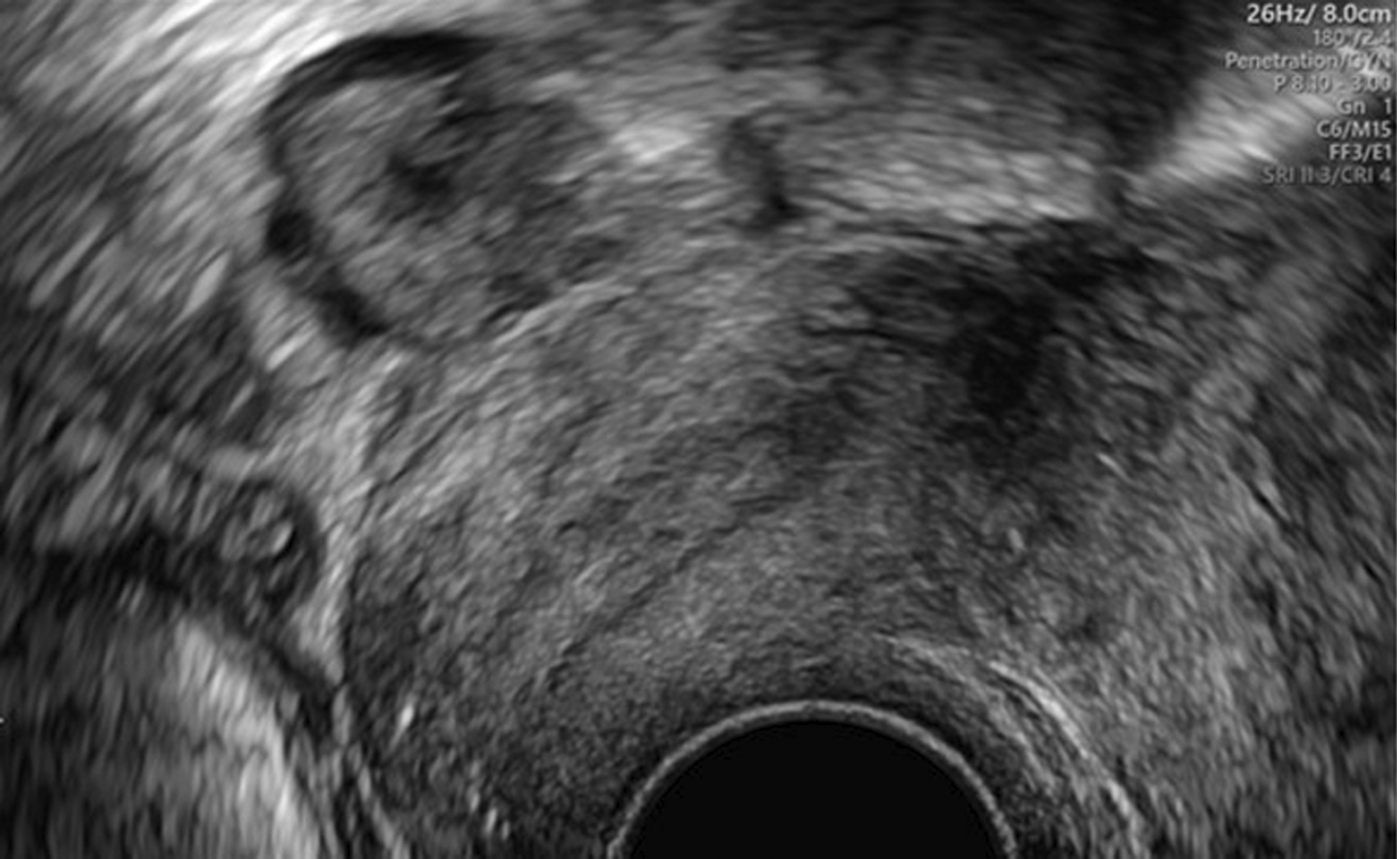
Transvaginal ultrasound image of abdominal ectopic pregnancy at 7 + 3 weeks' gestation, implanted at the pouch of Douglas.

**Figure 2:**
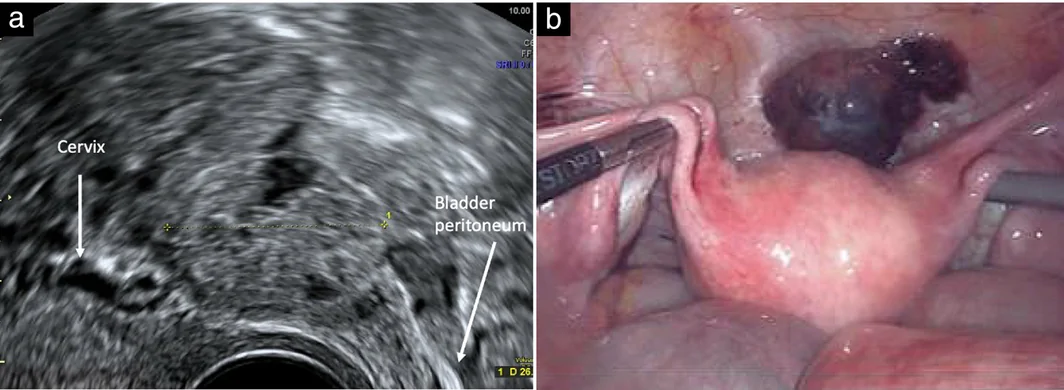
Abdominal ectopic pregnancy (AEP) at 6 + 1 weeks' gestation implanted at the uterovesical fold, visualized (a) on transvaginal ultrasound and (b) at laparoscopy. Calipers in (a) indicate diameter of AEP in one orthogonal plane.

**Figure 3:**
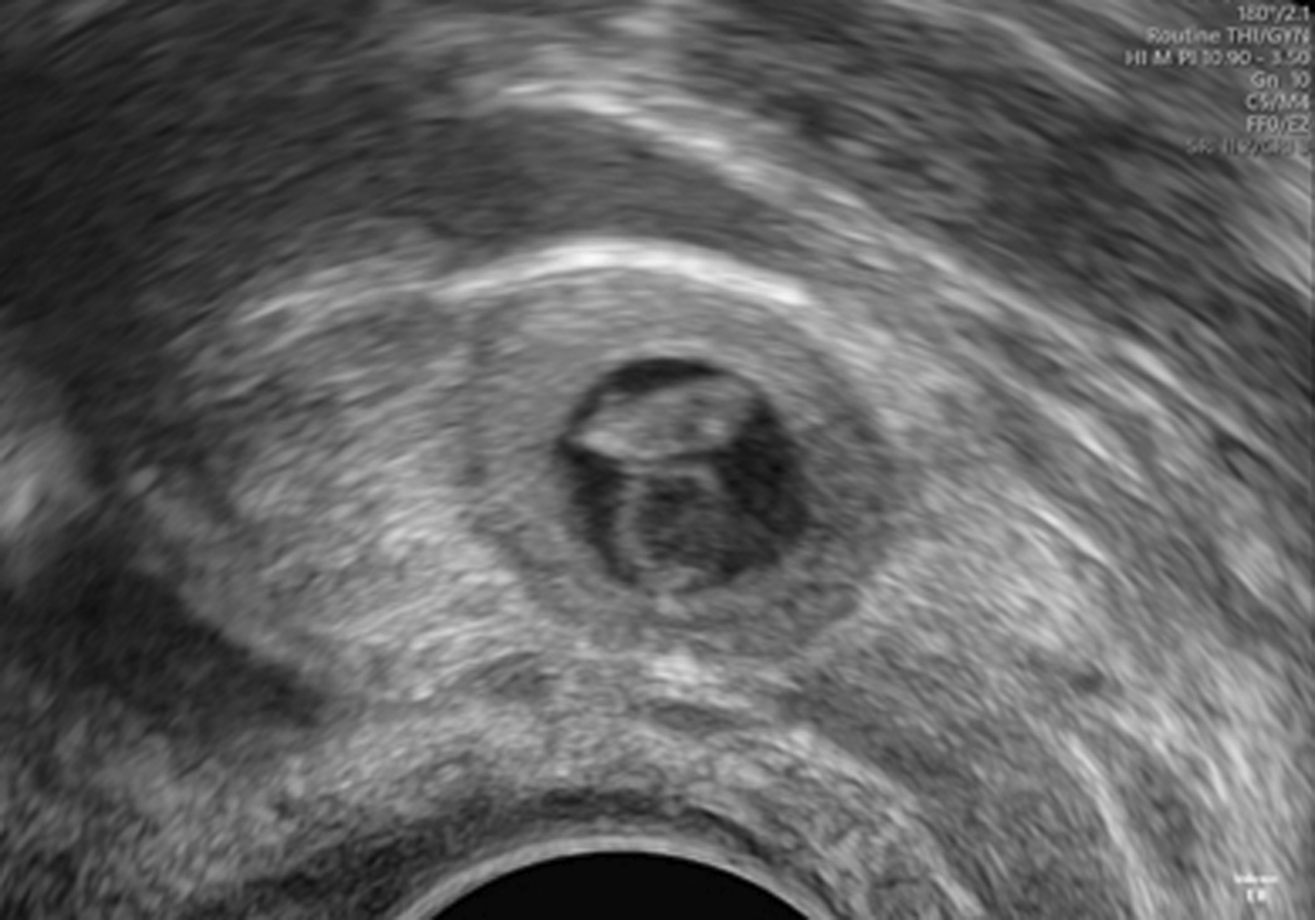
Transvaginal ultrasound image of abdominal ectopic pregnancy at 7 + 0 weeks' gestation, implanted at the left pelvic sidewall.

Various morphological features suggestive of AEP were identified on review of ultrasound images, which were available for 15 cases of first‐trimester AEP visualized pre‐operatively. These features are shown in Figure 4. All 15/15 (100%) pregnancies were visualized separate from the uterus and ovaries. Other than this, the most common morphological feature, present in 14/15 (93%) cases, was the absence of a hypoechogenic tissue layer surrounding the pregnancy. This is different from TEPs, which are generally surrounded by Fallopian tube. In 10/15 (67%) cases, the pregnancy was fixed to the peritoneum (negative ‘sliding organs’ sign) and in 11/15 (73%), color Doppler examination confirmed trophoblastic blood supply arising from the peritoneum. The finding of a negative ‘sliding organs’ sign was established by reviewing the ultrasound reports and discussing the findings with the operators who performed the original scans, where possible. There may have been additional cases with a negative ‘sliding organs’ sign, since it is not possible to confirm this finding retrospectively on still images. Among 15 first‐trimester AEPs, 1/15 (7%) demonstrated just one diagnostic feature, 1/15 (7%) demonstrated two diagnostic features, 5/15 (33%) demonstrated three diagnostic features and 8/15 (53%) demonstrated all four diagnostic features.

**Figure 4:**
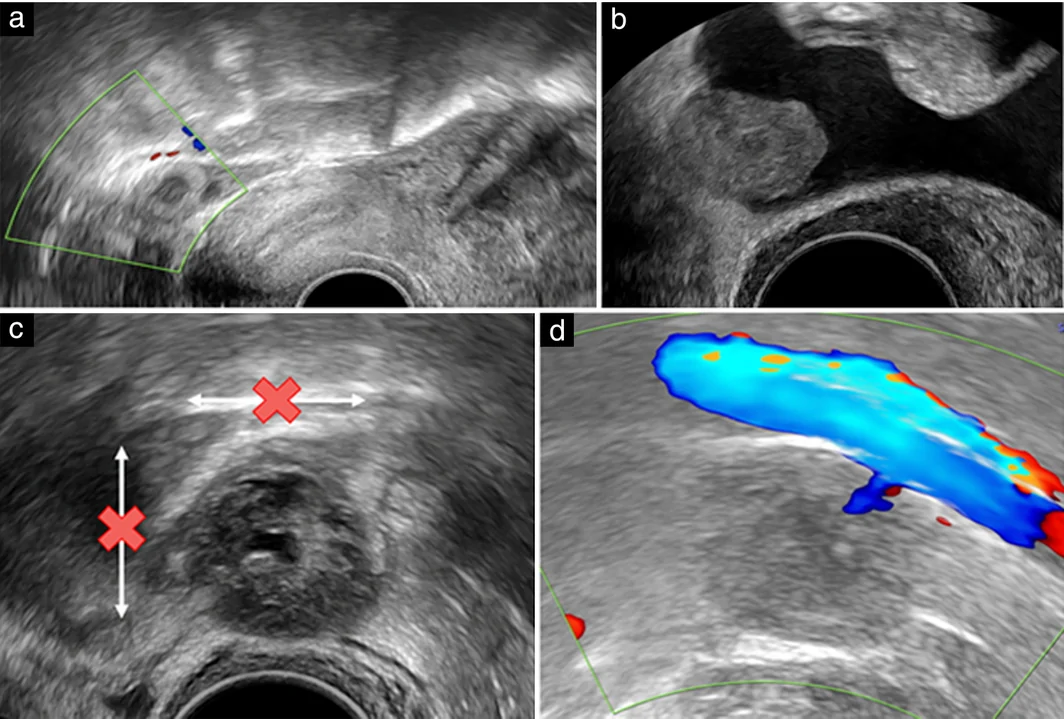
Transvaginal ultrasound imaging showing diagnostic features of early abdominal ectopic pregnancy (AEP), diagnosed in the first trimester: (a) pregnancy visualized separate to the uterus and ovaries; (b) absence of a hypoechogenic tissue layer surrounding the pregnancy; (c) negative ‘sliding organs’ sign; and (d) trophoblastic blood flow confirming peritoneal implantation site on color Doppler imaging.

### Treatment

The treatment and clinical outcomes are shown in Table 5. There was no significant difference in the success of expectant management or the overall proportion of patients that underwent surgery (either as their initial or final management) between the two groups.

**Table 5:** Clinical outcomes of abdominal ectopic pregnancy (AEP) and tubal ectopic pregnancy (TEP)

Outcome	AEP (*n* = 19)	TEP (*n* = 57)	OR (95% CI)	*P*	Adjusted OR (95% CI)*	*P*
Initial management†			NE	0.19	NE	—
Expectant	4 (21)	23/56 (41)	—		—	
Medical	0 (0)	2/56 (4)	—		—	
Surgical	15 (79)	31/56 (55)	—		—	
Final management†			NE	0.31	NE	—
Expectant	5 (26)	16/56 (29)	—		—	
Medical	2 (11)	1/56 (2)	—		—	
Surgical	12 (63)	39/56 (70)	—		—	
Successful expectant management	3/4 (75)	16/23 (70)	1.31 (0.12–14.93)	0.83	1.42 (0.12–17.20)	0.78
Surgery performed†	16 (84)	39/56 (70)	2.32 (0.60–9.04)	0.22	1.66 (0.38–7.29)	0.51
Operative blood loss ≥ 500 ml	4/16 (25)	1/36 (3)	11.67 (1.18–114.90)	0.04	NE	NE
Blood transfusion†	4 (21)	0/56 (0)	NE	0.003	NE	NE
Length of hospital stay (days)‡	2.9 ± 1.7	2.4 ± 0.7	0.6 (–0.1 to 1.2)	0.09	0.1 (–0.7 to 0.9)	0.73

Data are given as *n* (%), *n*/*N* (%) or mean ± SD, unless stated otherwise.

* Adjusted for previous termination of pregnancy, previous ectopic pregnancy and mode of conception.

† Data unavailable for 1/57 patients with TEP referred with persistent pregnancy of unknown location, who returned to their local hospital for management.

‡ Data for length of hospital stay are given as mean difference (95% CI), and were unavailable for 3/16 patients admitted with AEP and 1/39 patients admitted with TEP. NE, not estimable; OR, odds ratio.

Among controls with TEP who underwent surgery, 35/39 (90%) had salpingectomy, 3/39 (8%) had salpingotomy and 1/39 (3%) underwent removal of expelled pregnancy tissue without tubal surgery. Among cases of AEP that underwent surgery, 11/16 (69%) underwent successful laparoscopic excision of AEP. In 1/16 (6%) cases, conversion to laparotomy was required. This was the only case of AEP diagnosed after 12 weeks' gestation. In 4/16 (25%) cases, the pregnancy was not completely excised at laparoscopy. In 2/4 cases, the pregnancy was not immediately visible nor accessible at laparoscopy. In the first, intraoperative ultrasound was used to locate the pregnancy, excision of which would have required dissection in proximity to major vessels and the ureter. Instead, transvaginal ultrasound‐guided embryocide and local injection of methotrexate were administered. This was successful, with a hCG resolution time of 34 days. In the second case, the pregnancy was found to be surgically inaccessible owing to adhesions. Ultrasound‐guided local injection of methotrexate was planned but serum hCG levels fell spontaneously, so the patient opted for expectant management. This was successful, with a hCG resolution time of 31 days. In the remaining two cases, surgical excision was incomplete. One patient had an emergency laparoscopy for massive hemoperitoneum but was subsequently referred to our center with a residual ectopic pregnancy, which was managed expectantly. The other patient underwent salpingectomy for suspected TEP. Although histological analysis of the excised Fallopian tube confirmed TEP, an ectopic pregnancy remained visible on subsequent ultrasound assessments, reflecting an ongoing (secondary) AEP. This was diagnosed when the patient returned with pain and persistently raised hCG levels 3 weeks following surgery. The ultrasound findings were typical of retained pregnancy tissue, which was visualized separately to the uterus and ovaries, without an overlying tissue layer, and was fixed to the peritoneum, with peritoneal implantation confirmed on color Doppler examination. This case was managed with systemic methotrexate, which was successful with a hCG resolution time of 27 days.

Among patients who underwent surgical excision of AEP, the median hCG resolution time was 14 (IQR, 14–17) days. An unadjusted analysis suggested that significant operative blood loss (≥ 500 ml) was significantly more common in AEP, with the odds being 12 times higher than in TEP (4/16 (25%) *vs* 1/36 (3%); OR, 11.67 (95% CI, 1.18–114.90); *P* = 0.04). The need for blood transfusion was significantly higher in AEP than in TEP (4/19 (21%) *vs* 0/56 (0%); *P* = 0.003).

### Future pregnancies

At the time of writing, 6/19 (32%) patients had a known pregnancy following AEP. Three pregnancies were conceived spontaneously, resulting in live birth, and three pregnancies were conceived via IVF. Of the three pregnancies conceived via IVF, one resulted in live birth, one resulted in early miscarriage and one was ongoing at 8 weeks' gestation. At the time of writing, 21/57 (37%) patients had a known pregnancy following TEP. Fifteen pregnancies were conceived spontaneously, of which 14 resulted in live birth and one was a TEP. Six pregnancies were conceived via IVF and resulted in live birth. There was no significant difference in the rate of live eutopic pregnancies following AEP compared with TEP (5/6 (83%) *vs* 20/21 (95%); OR, 0.25 (95% CI, 0.01–4.73); *P* = 0.40).

## DISCUSSION

To our knowledge, this is the largest study to describe the clinical and sonographic features of early AEP and to propose specific ultrasound‐based diagnostic criteria. The majority of cases were classified accurately as an ectopic pregnancy at the initial ultrasound examination, however a third of all AEP cases were mistaken for TEP. Our findings suggest an association between IVF and AEP, making AEP an important consideration in early pregnancy scans after assisted conception. This study also shows that, when AEP is diagnosed in the first trimester, both expectant and surgical management may be utilized depending on clinical symptoms, ultrasound features, and hCG levels and dynamics. Ultrasound‐guided local injection of methotrexate is useful in surgically complex cases, such as when a pregnancy is covered by dense adhesions or when surgical removal carries a high risk of organ or major‐vessel injury.

The main limitation of this study is the modest number of cases, which reflects the rarity of AEP. The low event rate reduces the precision of the statistical effect estimates and contributes to the risk of overfitting and potential mathematical instability within the multivariable model. This requires the adjusted analyses to be interpreted with a degree of caution. Secondly, owing to the retrospective study design, some background data on ethnicity and body mass index are missing. The use of complete case analysis to handle missing data further reduces statistical power and may introduce bias if data were not missing at random. Controls were selected exclusively from the primary center, where 15/19 (79%) cases of AEP were diagnosed. Although 2/3 additional sites were also NHS early‐pregnancy units in London, this has potential to introduce bias due to potential for variation in patient characteristics between centers, which could have affected the estimates of association between IVF and AEP. Additionally, given that 1/3 of AEPs were misdiagnosed as TEP, and approximately 1/3 of controls with TEP were managed expectantly, it is possible that a proportion of controls were actually undiagnosed cases of AEP, albeit this is statistically unlikely given the difference in relative incidences of the two conditions. Larger prospective studies would be optimal to validate the suggested diagnostic criteria but may be difficult to accomplish given the rarity of AEP.

Our findings show that the symptoms of early AEP are non‐specific, which is in agreement with previous literature^10^, ^11^, ^12^, ^13^. Patients with AEP were significantly less likely to have vaginal bleeding than patients with TEP. This is in keeping with previous studies on non‐tubal ectopic pregnancy performed at our center, which have demonstrated a lower prevalence of early‐pregnancy vaginal bleeding in ovarian (35%) and intramural (44%) ectopic pregnancies^16^, ^17^ when compared with TEP.

On the other hand, our study demonstrates that high‐quality ultrasound is a reliable diagnostic tool for discriminating between AEP and other early‐pregnancy complications. We report an ultrasound detection rate of 67% for AEP, with the majority of cases diagnosed correctly at the initial ultrasound examination. This is higher than has been reported previously and suggests that AEP should be considered an ultrasound diagnosis, not a surgical diagnosis. In all cases in which an ultrasound diagnosis of AEP was not achieved, the pregnancy visualized was initially misclassified as TEP. Based on our findings, we propose novel sonographic criteria for the diagnosis of AEP. Separation of the pregnancy from other pelvic structures was required to differentiate AEP from eutopic, uterine ectopic and ovarian ectopic pregnancy. As such, the first criterion is the presence of a gestational sac or trophoblastic tissue that is separate from the uterus and ovaries. The absence of an overlying hypoechogenic tissue layer was critical in distinguishing AEP from TEP. The second diagnostic criterion is therefore the absence of a hypoechogenic tissue layer overlying the pregnancy. Peritoneal implantation was also suggested by a negative ‘sliding organs’ sign, which is positive in TEP owing to mobility of the Fallopian tube. This was assessed by using the non‐dominant hand to perform gentle abdominal palpation of the pregnancy against the transvaginal ultrasound probe; the third diagnostic criterion is therefore a negative ‘sliding organs’ sign. Finally, certainty of diagnosis of AEP can be achieved by identification of the blood supply arising from the fixed peritoneal surface. As such, the fourth and final diagnostic criterion is evidence of blood supply derived from the peritoneal surface on color Doppler examination. In 13/15 (87%) cases, at least three ultrasound features were present and all four features were present in 8/15 (53%) cases. Therefore, we propose that all ultrasound examinations of ectopic pregnancy include assessment for these four features. We suggest an ultrasound diagnosis of AEP is considered if any one feature is present and an ultrasound diagnosis of AEP is made if three or more features are present.

This study has identified a significant association between conception via IVF and AEP. Although assisted reproductive technology (ART) was previously considered a risk factor for ectopic pregnancy, a recent meta‐analysis demonstrated a reduction in incidence, reporting similar rates to those of the general population^18^. Given the relative incidence of different types of ectopic pregnancy, most patients included in the meta‐analysis had TEP, and therefore reflected our control group. The reduction in the association between ART and ectopic pregnancy over time is thought to relate to changes in the incidence of specific causes of subfertility and modifications to the practice of ART, such as fewer multiple‐embryo transfers. It is notable that in our study, half of the cases of AEP conceived via IVF occurred after double‐embryo transfer. The association between AEP and IVF does not necessarily implicate the process of IVF; it may be that biological attributes that predispose to AEP are overrepresented in the population undergoing IVF or being selected for double‐embryo transfer. Regardless of pathophysiological mechanism, given our findings, it is vital that clinicians are mindful of AEP when performing early‐pregnancy scans after assisted conception.

Mode of conception was the only factor associated with AEP that reached statistical significance. However, the multivariable analysis showed a non‐significant trend towards a lower rate of AEP with previous TOP and higher rate of AEP with previous ectopic pregnancy. In the univariable analysis, an association with endometriosis closely approached significance (*P* = 0.06) but the effect was not demonstrated after adjustment for previous TOP and previous ectopic pregnancy in the multivariable analysis. A potential interpretation is that TOP often occurs in the context of unwanted eutopic pregnancy, reflecting high fecundity, whereas previous ectopic pregnancy and endometriosis are more likely to be associated with pelvic adhesions or subfertility, which may also predispose to AEP. As well as potentially being a risk factor for AEP, pelvic adhesions may complicate surgical management of the condition. This was true for two cases in our study, in which the pregnancy was surgically inaccessible and reversion to expectant or medical management was required.

Early diagnosis of AEP is essential to reduce the risk of complications and minimize morbidity. Our study shows that when diagnosed during the first trimester, expectant management may be suitable for selected cases and when surgery is indicated it can be performed laparoscopically, whereas laparotomy was required for the only case diagnosed after 12 weeks' gestation.

### Conclusions

This study indicates a strong potential association between AEP and IVF, which is important to consider when assessing patients with a suspected or confirmed ultrasound diagnosis of extrauterine ectopic pregnancy. This finding could help to avoid misdiagnosing AEP as TEP in clinical practice. Most patients diagnosed with AEP require surgery, but expectant and medical treatment with methotrexate may be used in selected cases. We hope that the proposed refined sonographic criteria for AEP will help to facilitate earlier detection, which is essential to plan appropriate management and limit maternal morbidity.

## Data Availability

The data that support the findings of this study are available from the corresponding author upon reasonable request.
